# Coping with continuous human disturbance in the wild: insights from penguin heart rate response to various stressors

**DOI:** 10.1186/1472-6785-12-10

**Published:** 2012-07-11

**Authors:** Vincent A Viblanc, Andrew D Smith, Benoit Gineste, René Groscolas

**Affiliations:** 1 Département Ecologie, Physiologie et Ethologie (DEPE), Institut Pluridisciplinaire Hubert Curien (IPHC), Université de Strasbourg, 23 rue Becquerel, Strasbourg 67087, France; 2 CNRS, UMR7178, Strasbourg 67087, France; 3 Department of Ecology and Evolution, Biophore, University of Lausanne, Lausanne CH-1015, Switzerland

**Keywords:** Stress, Heart rate, Habituation, Selection, Seabird, Human disturbance, Long-term monitoring

## Abstract

**Background:**

A central question for ecologists is the extent to which anthropogenic disturbances (*e.g.* tourism) might impact wildlife and affect the systems under study. From a research perspective, identifying the effects of human disturbance caused by research-related activities is crucial in order to understand and account for potential biases and derive appropriate conclusions from the data.

**Results:**

Here, we document a case of biological adjustment to chronic human disturbance in a colonial seabird, the king penguin (*Aptenodytes patagonicus*), breeding on remote and protected islands of the Southern ocean. Using heart rate (HR) as a measure of the stress response, we show that, in a colony with areas exposed to the continuous presence of humans (including scientists) for over 50 years, penguins have adjusted to human disturbance and habituated to certain, but not all, types of stressors. When compared to birds breeding in relatively undisturbed areas, birds in areas of high chronic human disturbance were found to exhibit attenuated HR responses to acute anthropogenic stressors of low-intensity (*i.e.* sounds or human approaches) to which they had been subjected intensely over the years. However, such attenuation was not apparent for high-intensity stressors (*i.e.* captures for scientific research) which only a few individuals experience each year.

**Conclusions:**

Habituation to anthropogenic sounds/approaches could be an adaptation to deal with chronic innocuous stressors, and beneficial from a research perspective. Alternately, whether penguins have actually habituated to anthropogenic disturbances over time or whether human presence has driven the directional selection of human-tolerant phenotypes, remains an open question with profound ecological and conservation implications, and emphasizes the need for more knowledge on the effects of human disturbance on long-term studied populations.

## Background

Whereas considerable knowledge in ecology and animal behaviour has been gained from scientific research on wild animal populations (*e.g.*[1-17], reviewed in [18]), continuous exposure to humans can have profound effects on the biology of wild species, *e.g.*[15,19-21]. Thus, a crucial aspect of ecological research is to investigate and identify those effects (especially that of chronic disturbance), in order to understand and account for potential biases when deriving conclusions from the data yielded by studies in the wild [17]. Several authors have questioned how the exposure to anthropogenic disturbance might affect the biology of species under study [19-21]. For instance, some species have been shown to habituate to (*i.e.* tolerate) [22] frequent human disturbance (*e.g.* marine iguanas, *Amblyrhynchus cristatus*; [19]; Magellanic penguins, *Sphenicus magellanicus*; [20,23]; Jackass penguins, *Spheniscus demersus*; [24]). In contrast, other species have been shown to sensitize to human stressors (*e.g.* Yellow-eyed penguin, *Megadyptes antipodes;*[25]), and others still have been shown to exhibit different responses depending on their developmental stage (*e.g.* nestling or juvenile hoatzin chicks, *Opisthocomus hoazin;*[26]). Frequent anthropogenic disturbance is also known to drastically alter behaviour patterns, *e.g.* in threatened killer whales intense boat trafficking results in a 14% decrease in the animals’ foraging time [21], and to affect reproductive output [25,27], or offspring provisionning [28].

A major complication of assessing the consequences of human disturbance on wildlife, is that those consequences are not always directly visible. For instance, even if seemingly unaffected (*i.e.* behaviourally calm), animals might undergo profound physiological changes in response to anthropogenic disturbances, or even to the mere presence of human observers (*e.g.* changes in heart rate, [29-32]).

So what can be said about the continuous presence of humans in specific wildlife populations for the purpose of long-term monitoring and scientific research? To what extent do researchers affect natural processes? There is a need for more data in order to evaluate the impacts of anthropogenic disturbances on wildlife, especially for protected species in pristine environments. Such studies are essential not only because they enable to establish guidelines for the conduct of scientists towards studied species and the management of tourism and recreational activities in natural habitats [26,29-34], but especially because of their implications on the way we think about scientific experiments in the wild, and the inferences we derive from those experiments [17].

**Figure 1:**
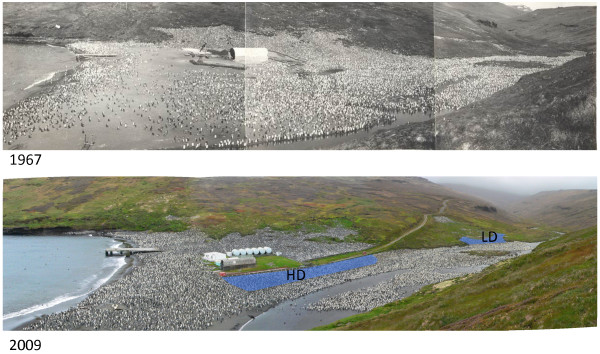
** Chronic human disturbance in the king penguin colony of ‘La Baie du Marin’ on Possession Island (Crozet Archipelago, 46°25’ S, 51°45’E).** In 1961–1962, a first camp was installed on the beach side of the colony (top picture taken in 1967). Since then, logistic facilities have remained and scientific facilities have been installed at the same place. After 1967, a road was built in order to facilitate logistic operations and transits of material (food, equipment) to the permanent station situated some 500-m above (bottom picture). Thus, part of the colony has been subjected to the regular presence of humans and their activities (scientific or other), whereas another part far from the facilities was left relatively undisturbed. This study is based on the comparison of the HR stress response of birds located within the areas of high (HD) or low (LD) human disturbances (blue-shaded areas). Photo credits: Archive biology lab, Crozet/IPEV. Claire Saraux/IPEV.

Here, we report a case of biological adjustment to human disturbance in a wild king penguin (*Aptenodytes patagonicus*) colony of the Crozet Archipelago, which has been exposed to the continuous presence of humans for over 50 years. In 1961–1962, a permanent camp was established on Possession Island [35] (Figure 1 top) both within and close to one of its major king penguin colonies: the ‘Baie du Marin’ (BDM) colony. As part of an international scientific effort to understand polar ecosystems, research in this penguin colony has been on going since the early 60’s. This has provided us with a unique opportunity to investigate the effects of continued human presence on the physiology of breeding penguins. We specifically question how breeding king penguins cope with chronic anthropogenic disturbance and consider whether heart rate (HR) responses to acute human stressors may be influenced by a history of close contact with humans. Using HR-loggers (see [36]) to monitor the stress response of penguins, we tested whether HR responses differed between birds holding breeding territories in colony areas subjected to very frequent (daily or more, see methods) human disturbance and birds breeding in relatively undisturbed (weekly or less) areas. Three different acute human stressors were applied, *i.e.* a loud metal sound, a distant approach, and a capture. In the BDM colony, loud metal sounds typically occur during the logistic operations that take place close to disturbed areas several times a year (*e.g.* cranes and trucks used during stevedoring for Island supply). Distant approaches occur when scientists/tourists observe birds from the edges of the colony, whereas a limited number of captures are performed annually by scientists for research. HR provides a highly sensitive measure of stress responses, that may be modulated independently of hormonal pathways [37] and allow greater insight than hormonal responses (such as corticosterone) on how stress responses are shaped depending on the specific nature of various stressors [37,38]. Using this method gave us the possibility: (i) to investigate how stress responses were shaped by chronic exposure to humans, and (ii) how this shaping might have varied according to stressor type and intensity, and potential risk for the animal. 

## Methods

### Study population and location, bird marking and pre-disturbance follow up

Fieldwork was conducted on Possession Island, Crozet Archipelago (46°25’S, 51°45’E) in February-March 2011. Penguins belonged to the BDM colony, which is host to over 24 000 breeding pairs. This colony is located in the vicinity (some 500 m) of a permanent station (Alfred Faure) and is adjoining a beach that has been regularly used for logistical operations over the last 50 years, and where scientific shelters and technical buildings have been installed (Figure 1).

Twenty pairs of king penguin were randomly selected from the colony and flipper-banded at the onset of incubation (semi-rigid P.V.C. Darvic bands; 25.8 mm wide, 1.9 mm thick, 7.4 g) to allow their identification and follow-up. This size sample complied with permits to manipulate birds in the BDM colony (see below). Ten pairs were located in a part of the colony adjoining permanent buildings and also very close (5–10 m) to a permanent road used daily by pedestrians and in some occasions by motorised vehicles (Figure 1: HD-area). Throughout the year and over the last 50 years, this part of the colony has been visited daily at a short distance (< 5 m) by at least one human, and in some occasions by several groups of up to 10 visitors over a day. In addition, this is also the part of the colony where intensive scientific research has been conducted over the last 20 years, which implied approaching/entering the colony several times a day, including for bird capture purposes, almost all year-round. The other ten pairs were located in a remote part of the colony (about 300 m away from the beach; Figure 1: LD-area), not exposed to anthropogenic noises and where human visitations were much less frequent (around one visit per week over the last 10 years).

Due to time constraints with fieldwork, we subjected 33 out of the 40 birds to three types of acute stressors (see below) 50–80 days after banding. Males king penguins start to incubate after the female has laid their only egg, and partners subsequently alternate between incubation/brooding duties on land and foraging trips at sea throughout the season [39,40]. The specific breeding phenology of king penguins allowed us to determine the date of the onset of each incubation and brooding shift (mean duration of 15 and 12 days for incubation and brooding shifts, respectively), and to ensure that all birds (females at shift 4 and 6 of breeding, and males at shifts 5 and 7) used in this study were in a similar breeding status: birds brooding a non-thermally emancipated chick aged from 2 days to 1 month. This was important, as animals may perceive specific stressful stimuli differently depending on their life-history stage. In addition, stress responses may also be under seasonal variation [41,42]. Comparision of responses should thus be made within life history stages [42]. Eighteen of the stressed birds were located in the part of the colony with a very low rate of chronic human disturbance (the LD-area) and fifteen of them were in the part subjected to a very high rate of chronic disturbance (the HD-area).

### Heart rate monitoring

Prior to being stressed and usually within 3 days after the onset of a brooding shift, penguins were equipped with externally mounted HR-loggers (Polar® model RS800, Polar Electro Oy, Kempele, Finland), within the colony and on their breeding territory (see details in [36] for equipment, logger technology and accuracy of HR measurement). Each bird was equipped only once. At capture, the bird’s head was cover with a hood to keep it calm. The logger transmitter (weighing less than 1% of total body mass) was attached to the middle of its back with Tesa® tape, and the receiver fixed on a metal pole within a 5-m distance of the animal. Such a set up prevented the equipment from hindering the movements of the birds. This was confirmed by the fact that we never observed birds trying to remove electrodes or transmitters, nor did we observe any adverse effects of the equipment on the birds’ health or behaviour. Most animals developed a tachycardia due to handling (up to 165 beats per minute on average), from which they usually recovered within 15–30 min following release. Handling lasted between 5 and 10 min and this procedure never resulted in chick abandonment. Birds resumed normal activity (*i.e.* resting, comfort behaviour or aggressive interactions with neighbours) within minutes after release. HR-loggers were set to store the sampled data for up to 3 days and sampling was set at a rate of one data point every 2 seconds. Following equipment, birds were left to recover for at least 12 h, *i.e.* one night, before stressors were applied. We retrieved all equipment from the birds 2–3 hours following the last stress protocol. It is important to note that all individuals in this study were manipulated for a similar amount of time before stressors were applied. Differences in HR stress responses are thus not likely to be related to any prior manipulation undergone in order to band the birds and deploy the HR-loggers.

### Stress protocols

**Figure 2:**
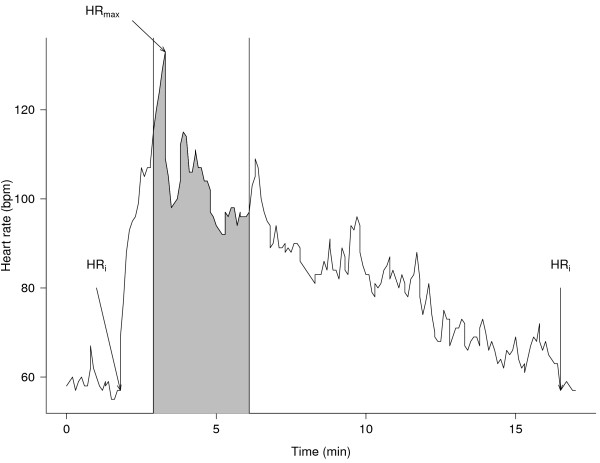
** Typical heart rate (HR) response of a brooding king penguin to a human stressor.** In this case, the bird was submitted to a capture-immobilization stress, being approached, captured and held captive for 3 minutes (grey area; see text). The approach occurred out of the grey area. HR_i_ is the initial HR before HR started to increase in a constant way, and the HR reached after full recovery; HR_max_ is the maximum HR reached during the stress. HR is expressed in beat per min (bpm).

Three different acute stressors were applied in a standardized manner to each bird: a human approach up to 10 m from the bird, a capture-immobilization and a sound. The approach and sound stressors were chosen as representative of those to which penguins are regularly submitted in the part of the colony with a high rate of human disturbance, the capture stress being in contrast only occasionally applied to few individuals. Stressors were applied in a random order, over two days and with at least 5 hours separating stressors. The order in which stressors were applied did not affect the corresponding HR response (LMMs; *t* = 0.60 and 1.17, *p* = 0.55 and 0.24, *n* = 76, *N* = 33 birds; for HR excess and maximum HR increase, respectively). Observations on the focal bird at a *ca.* 30–35 m distance started several minutes before stressors were applied to ensure that it was not sleeping and thus could both see and/or hear the experimenter or the sound, respectively. Moreover, we ensured that the birds were in a resting state for several minutes before proceeding with the test so that they maintained a baseline HR (see Figure 2). While stressing the birds, their behaviour and the distances from which the experimenters found themselves from the focal subject (estimated visually after training) were recorded in real time using a digital audio recorder (VN5500® Olympus Europa, Hamburg, Germany). Behavioural observations continued several minutes after the stressor was applied. These observations were done in order to account for the potential effect of routine bird behaviour on HR, *i.e.* physical activities (aggressive interactions with neighbours, comfort behaviour, chick care or feeding). Indeed, physical activities not directly related to the stress response risked inducing significant HR increases, and thus bias the calculation of some parameters allowing us to characterize the response to a given stressor. The specific protocols for each type of stress were as follows:

#### 10-m approach stress

Penguins were approached from the front from a starting distance of at least 30 m within the bird’s visual field and at an average speed of 0.5 m/sec. The starting distance was chosen from preliminary tests showing that in the BDM colony, the physiological detection distance of penguins when approached by humans (*i.e.* the distance at which HR started to increase) was around 20–25 m. Thus at 30 m, birds did not exhibit behavioural signs of vigilance towards the experimenter and HR remained at resting levels. The experimenter stopped 10 m away from the bird where he remained motionless for 1 min (to standardize the approach and mimick a standing observer at the edge of the colony) while dictating observations on the behaviour of the subject, then subsequently retreated at a constant speed. This distance was chosen because preliminary tests showed that king penguins breeding in the BDM colony become behaviouraly alert when approached by humans from a distance of 10 m or less (Groscolas & Viblanc, *unpublished data*).

#### Capture-immobilization stress

The protocol was the same as for 10-m approaches except that the focal bird was approached until capture, which was eased by the fact that brooding penguins have a chick in their brood pouch and cannot escape rapidly. Upon capture, the bird was gently immobilized for 3 minutes, its head covered with a hood. The hood was then quickly removed and the experimenter retreated at a constant speed to the original position, some 30 m away from the animal, in order to continue behavioural observations for several minutes.

#### Sound stress

Birds were discreetly approached from behind until the experimenter was 15 m behind them, but not sighted. After the bird was observed resting for at least 3 min, the experimenter struck two hollow metal bars three times with a 1-sec interval. The magnitude of the noise averaged 102.5 ± 0.3 dB (n = 100 measurements), *i.e.* a magnitude sufficient enough to be alarming to a bird [43], and assumed to be similar in intensity to metal sounds that might occur when machines are operating close to the colony (during stevedoring operations).

### Heart rate analysis

HR data were expressed in beat per min (bpm), plotted and analysed using Polar Pro Trainer® v.5.00.105 software. Audio recordings of each test were time-matched (by previous synchronization of the observer’s digital watch with that of the HR-logger at ± 1 sec.) with the corresponding HR data, which allowed to calculate a number of parameters describing the subjects’ HR responses to the stress (Figure 2). The duration of a HR response was characterized as the total time that HR was elevated above the initial resting rate (HR_i_), *i.e.* from HR starting to increase until recovering to initial level. We defined HR_i_ as the HR at the moment preceding a rapid constant increase in HR. Maximal HR (HR_max_) achieved during the stress was determined and relative maximal increase in HR (in %) was calculated as: 100 * (HR_max_ – HR_i_)/HR_i_. We also calculated excess HR, *i.e.* the number of heart beats produced in excess of resting HR due to stress, as (mean HR during stress – HR_i_)*duration of HR elevation (in min). Thus, excess HR (in beats) approximated the area under the HR curve and above resting values. We defined HR reactivity as the maximal increase in HR/time needed to reach the maximum HR, *i.e.* a speed of HR increase from HR_i_ to HR_max_. Similarly, HR recovery was defined as the speed of HR return to HR_i_ following the stress (*i.e.* from HR_max_ to resting levels again, in bpm/sec). In some cases and mostly following capture-immobilization, the HR profile during the recovery period was affected by interfering unrelated behaviour and physical activity. We discarded such cases, so that the actual sample size in final calculations is lower than the number of stressed birds (of 33 stressed birds, only 28 sound, 28 10-m approach and 20 capture stresses were retained).

### Statistics

All statistical analyses were performed using R v.2.10.1 [44]. As each individual was only tested once for each stressor, data was analysed with linear regression models (LMs) when stressors were considered separately. Linear Mixed Models (LMMs) were used when stressors were pooled, and bird identity was then specified as a random factor, *i.e.* up to 3 repetitions (one sound stress, one 10-m approach stress, and one capture stress) per individual bird. LMMs were performed using the ‘lme’ function of the ‘nlme’ package in R [45]. Residual normality was asserted using the Shapiro-Wilk normality test. Wherever necessary and to ensure normality of residuals was satisfied, data was transformed prior to analysis using Box-Cox power transformations [46], *i.e. x’ = (xp –1)/p,* where *p* is the power maximizing normality likelihood obtained with the ‘bcPower’ function from the ‘car’ package in R. Visual inspection of the residuals indicated no violation of assumptions of homoscedasticity. Significant values are reported for *p* < 0.05. *N* and *n* represent the number of stressed birds and of stresses, respectively.

### Ethical note

We removed flipper bands from all banded birds following retrieval of equipment, as detrimental long-term effects of flipper bands are known to occur in king penguins [17,47]. Capture, banding and equipment procedures were all approved by the Ethical Committee of the Institut Polaire Français – Paul-Emile Victor. Authorizations to enter the colony and to manipulate a limited number of birds (from 20 pairs) were obtained from Terres Australes et Antarctiques Françaises. The experiments comply with the current laws of France.

## Results

**Figure 3:**
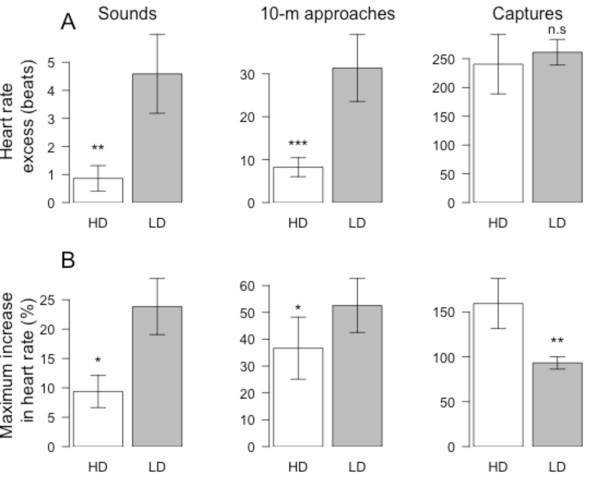
** Heart rate (HR) responses to 3 different types of human stressors (sound, 10-m approach, and capture-immobilization) for king penguins brooding in areas of high (HD) or low (LD) human disturbance.** (**A**) Excess HR is given in beats. (**B**) Relative maximum increase from resting HR (HR*i*) is given in percentage. Results are given as means ± SE. Statistics are figured for ^*^p < 0.05, ^**^p < 0.01, ^***^p < 0.001, ^n.s^Non-significant.

Overall, pooling all data together and controlling for stressor type by including it as a factor in the model, we found that brooders situated in an area of frequent human disturbance generally exhibited lower HR responses than their congeners breeding in an almost undisturbed area (LMMs; *t* = 4.3, *p* < 0.001, *n* = 76, *N* = 33 birds, and *t* = 2.07, *p* = 0.04, *n* = 76, *N* = 33 birds; for HR excess and maximum HR increase, respectively). However, this pattern varied depending on the type of stressor considered (*i.e.* the interaction between stress type and colony area significantly improved the models; *χ*^*2*^ = 6.05 and 12.49, *p* = 0.048 and 0.002; for HR excess and maximum HR increase, respectively), and also depending on the parameter used to calculate the HR response (Figure 3).

**Figure 4:**
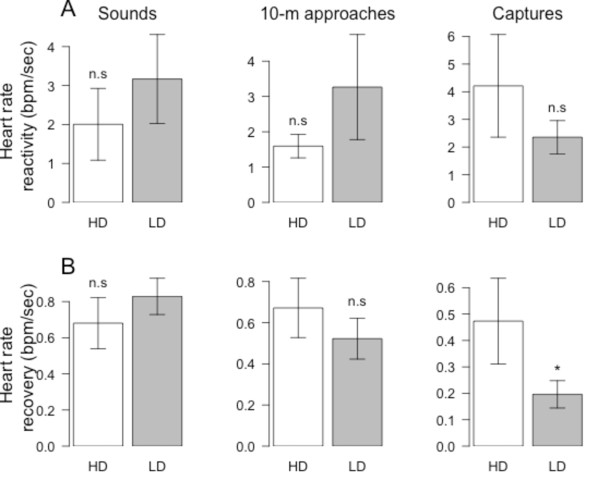
** Heart rate (HR) reactivity to, and recovery from, 3 different types of human stressors (sound, 10-m approach, and capture-immobilization) for king penguins brooding in areas of high (HD) or low (LD) human disturbance.** (**A**) HR reactivity (in bpm/sec) is the speed of HR increase to its maximum during the stress. (**B**) HR recovery (in bmp/sec) is the speed of HR decrease back to resting levels following reaching of HR*max*. Results are given as means ± SE. Statistics are figured for ^*^p < 0.05, ^n.s^Non-significant.

During sounds and 10-m approaches, HR excess was significantly 81% and 74% lower for birds breeding in areas of frequent human disturbance (LMs; *F*_1,26_ = 8.9, *p* = 0.006, *N* = 28 and *F*_1,26_ = 15.5, *p* < 0.001, *N* = 28; for sounds and 10-m approaches, respectively; Figure 3A). However, HR excess did not differ significantly between areas for captures (LM; *F*_1,18_ = 0.2, *p* = 0.669, *N* = 20; Figure 3A). Maximum relative increase in HR during sounds and 10-m approaches were also significantly 61% and 30% lower for birds breeding in areas of frequent disturbance (LMs; *F*_1,26_ = 6.5, *p* = 0.017, *N* = 28 and *F*_1,26_ = 4.3, *p* = 0.049, *N* = 28; for sounds and 10-m approaches, respectively; Figure 3B). In contrast, maximum relative increase in HR was actually 42% higher for birds in areas of frequent human disturbance when considering capture stresses (LM; *F*_1,18_ = 9.0, *p* = 0.007, *N* = 20; Figure 3B). The smaller HR excess observed both for sounds and 10-m approaches in birds breeding in areas of frequent human disturbance were not only due to a smaller maximum relative increase in HR but also to a much shorter duration of this increase. Indeed, this duration was 48% shorter for sounds (14.4 ± 2.6 sec *vs.* 27.8 ± 5.4 sec; LM; *F*_1,26_ = 4.4, *p* = 0.046, *N* = 28) and 52% shorter for 10-m approaches (51.1 ± 7.7 sec *vs.* 105.3 ± 12.2 sec; LM; *F*_1,26_ = 13.1, *p* = 0.001, *N* = 28). For captures, the duration of HR increase was also 38% shorter (376.3 ± 46.8 sec *vs.* 606.7 ± 83.3 sec), though not significantly (LM; *F*_1,18_ = 3.7, *p* = 0.070, *N* = 20) for birds in areas of frequent disturbance, explaining that despite a greater maximum relative HR increase, HR excess did not differ between the two areas. Whatever the type of stress, differences in HR response between the two colony locations were not due to differences in the HR reactivity (Figure 4A), nor to differences in HR recovery in the case of sounds and 10-m approaches (Figure 4B). It is interesting to note that following captures however, HR recovered much faster for birds located in areas of frequent disturbance compared to birds in undisturbed areas (Figure 4B).

## Discussion

We investigated the effects of chronic human disturbance on wildlife stress physiology. Working in a wild king penguin colony, areas of which have been exposed to continuous human presence for over 50 years, we found HR responses of breeding birds to acute human stressors to vary depending on (i) stressor intensity (and potential associated risk for the animal) and (ii) the frequency to which birds have been subjected to stressors over the years. Our results suggest that in highly disturbed (HD) areas, penguin HR stress responses to frequent and potentially innocuous stressors (such as loud sounds or distant human approaches) have been attenuated compared to undisturbed areas, whereas this was not the case for infrequent (and potentially noxious) stressors such as captures. Two hypotheses might explain our results: 1) physiological adjustment to continuous human disturbance and innocuous stimuli, *i.e.* habituation, or 2) behavioural desertion of the highly disturbed areas by the more stress-sensitive individuals, *i.e.* selection.

### Habituation or selection?

Our results suggest that the HR stress responses of king penguin in the BDM colony have been shaped according to the specific nature of the stressors they are subjected to. Indeed, whereas HR responses to sounds and 10-m approaches were attenuated in HD areas compared to LD areas, this was not the case for HR responses to captures, suggesting that attenuation was not a generalized phenomenon. Those differences are likely reflective of physiological habituation of breeding penguins to innocuous and repeated stimuli. Indeed, as comprehensively reviewed by Cyr and Romero [42], physiological habituation is likely to occur when an animal is repeatedly subjected to a specific innocuous stressor [42,48]. The intensity of stress responses to that particular stimulus may then decrease as the animal learns to consider the stimulus as innocuous [42]. It is important to understand that, in habituation, stress pathways are not blunted. Rather, the animal may learn to ignore the innocuous stimulus [42,49,50]. Hence, the phenomenon of habituation should remain stressor-specific not causing changes in the entire stress physiology of the organism: the capacity to respond to a novel stressor should remain unaffected [42]. This may be the case in our study, where HR responses were attenuated in HD areas for sounds and 10-m approaches, but not for captures. In our study colony, the degree to which birds have been exposed to the different stressors over the past 50 years is indeed very different. Whereas all birds in the HD area have been (and still are) regularly subjected to (potentially innocuous) approaches of human observers (whether scientists in the colony, technicians, or tourists on the outskirts) and anthropogenic sounds (*e.g.* machine noises during logistic operations), only a very limited number of individuals in each year are concerned by (potentially highly noxious) captures, which are exclusively conducted for scientific purpose. For instance, as a rough figure, one could estimate that during the Austral summer (when most of the scientific field work, logistic operations and tourist activity occur), the 3000–4000 birds in the HD area are approached by human observers 3–5 times per day. Over the course of the breeding season (*ca.* 4 months of intensive field work), this would amount to *ca.* 450*–*750 approaches (between 1 and 20 m) per bird, an estimation which is likely conservative. In stark contrast, captures in the HD area only concern some 50 individuals each year, which are caught and handled 1 to 5 times during the breeding season. In other words, one might consider that prior to our study, all the animals of the HD area had been subjected to very frequent human disturbance (*i.e.* anthropogenic sounds and approaches by human observers) over the years, whereas the likelihood that they had previously been captured and manipulated for scientific research is extremely weak. In addition, the intensity of the 3 stressors was certainly different, being low for sounds and 10-m approaches, and high for captures. Consistent with the idea that weak stimuli are more likely to result in pronounced habituation than strong stimuli [42,51], those results suggest that king penguin in the BDM colony may have habituated to repeated and potentially non-noxious stressors (sounds and 10-m approaches), but not to infrequent and potentially highly noxious stressors such as captures.

Previous studies have reported similar attenuation of stress responses to human disturbance in other species, *e.g.*[19,20,52]. In magellanic penguins for instance, birds nesting in HD areas showed lower behavioural and physiological responses to human visitation (*i.e.* tourist approaches) than birds nesting in LD areas [20,23,52]. However, it is interesting to note that in this case, attenuation of stress responses also extended to capture/restraint protocols, and adrenal responsiveness to ACTH injections appeared blunted in birds from HD areas [20]. This suggests that contrarely to king penguin, magellanic penguins had not actually habituated to human disturbance, but rather, that stress pathways were desensitized [42]. Could physiological desensitization have occurred for the king penguins in our study, so that stress responses would be attenuated in HD birds although sounds and approaches were still considered as stressful? The fact that stress responses remained unimpaired for captures suggests not. Taken together, those results emphasize the importance of considering species-specific responses to various stressors to fully understand how animals adjust to human disturbance.

Furthermore, our findings raise the question of whether HR attenuations in HD areas are actually the result of penguin habituation to innocuous stimuli, or whether they are the result of a selection on less stress-sensitive phenotypes. In other words, have stress-susceptible birds deserted highly disturbed areas over the years? This question is especially relevant as the existence of different animal temperaments and coping styles (*i.e.* animal personalities) is now widely supported [53-56], and variation in individuals’ temperament (*e.g.* human-tolerant phenotypes, [57]) has recently been suggested as an important factor to account for when analyzing the stress/behavioural responses of wildlife to human disturbance [32,58,59]. In line with this, HR responses of yellow-eyed penguins (*Megadyptes antipodes*) to a standardized human disturbance were found to vary depending on individual differences in temperament, and individual penguins were found to exhibit consistent HR responses over different breeding seasons, indeed suggesting that some personalities may be more stress-prone than others [32]. In addition, the spatial distribution of Eastern chipmunks (*Tamias striatus*) burrows in regards to human disturbance was found to be non-random, but rather dependent on individual temperament [59]. Although current data suggests there is some amount of intra-individual consistency in stress responses in king penguins (Viblanc, Smith, Gineste & Groscolas, *unpulished data*), we cannot conclude whether the observed differences in HR responses between LD and HD areas are reflective of individual differences in temperament or not. Nonetheless, marked intra-individual consistencies to human disturbance (*e.g.* flight initiating distance, heart rate stress responses) have previously been reported in birds [57] (including penguins [32]), which suggests that behavioural/physiological flexibility to human disturbance may be constrained by individual susceptibility to disturbance. Whether this may also be the case for physiological responses to human disturbance in king penguins remains to be explicitly tested. Assuming bird temperament may be heritable (*e.g.*[60,61]), this could be done by investigating physiological responses to acute stressors during early life-stages, *i.e.* chicks/juveniles, which have not long been exposed to human anthropogenic disturbances. If selection explains the pattern we observe in adults, one would expect chicks/juveniles to exhibit lower HR stress responses in highly-disturbed locations compared to chicks/juveniles in undisturbed areas. On the other hand, if birds have habituated to human disturbance over time, similar responses in chicks/juveniles should occur regardless of their location in the colony. Alternately, long-term records of breeding site fidelity may provide useful data to investigate territory distribution as a possible result of individual susceptibility to disturbance. Future studies might, for instance, consider monitoring the behaviour and physiological stress responses of marked individuals over the years in relation to their location in the colony.

### Implications for the study and conservation of wild populations: *Pros* and *cons*

Studies that have considered the effects of human disturbances on the biology of various species have focused especially on the (detrimental) effects of tourism and industry on wildlife, *e.g.*[21,23-28,62,63]. Along with the massive explosion of ecotourism to even the most remote parts of our planet (*e.g.* Antarctica, [64]), such studies have been essential in assessing the impact of human activities on wildlife in order to establish guidelines for conservation purposes [27-29,65]. One of the pitfalls of such research is perhaps to forget that from the perspective of wildlife, tourism and scientific research are not two worlds apart. Long-term scientific research programs might also have profound effects on wild populations, *e.g.* [17, 47, this study]. The question is then whether those effects are detrimental or not to the species and studied population. As challenged by Nisbet [22], human (and researcher) activity may only be considered a disturbance if it is shown to adversely affect species fitness, *e.g.* breeding success, survival, population decline. Physiological effects of human activity (such as changes in hormone concentrations, HR), may thus not necessarely qualify as adverse, unless they are actually shown to decrease fitness [22]. At our study site, habituation to innocuous stressors such as sounds or the presence of human observers may on the contrary be beneficial to scientific research, as birds decrease the amount of energy invested in costly stress responses, learning to ignore the lurking scientist observing them with his/her binoculars and talking into his/her tape-recorder – habituation is, after all, adaptive by definition. Nonetheless, understanding the consequences of scientific research (*e.g.* attaching measuring devices, long-term monitoring) [17,66-69] on animal behaviour and physiology is essential in setting-up experiments and protocols, and drawing conclusions from the data collected. In this regard, reports documenting the effect of anthropogenic agents on wildlife physiology are needed, as it is only through such knowledge that researchers may draw un-biased conclusions from studies in the wild [67,69]. For instance, as in the case of the king penguins from the BDM colony, it is important to be aware of potential differences in animal sensitivity to human researchers according to various areas of the colony. Animal populations are likely to vary in terms of how intensely parts of the population are disturbed by anthropogenic agents, *e.g.*[19,20], so that generalized conclusions on whole populations or species may be inapropriate if derived from a biased sample. Further still, as discussed above, if chronic human disturbance is indeed selecting for less stress-sensitive individuals, this could have strong implications in terms of conservation. Human disturbance is an important driver of directional phenotype selection [70], and selective desertion of the more stress-sensitive phenotypes in specific populations could lead to a loss in phenotypic plasticity and/or genetic diversity [70]. In turn, this may render chronically disturbed colonies less flexible to environmental change, *e.g.* climate.

## Conclusion

Our findings report a case of physiological adjustment to human presence in a long studied king penguin colony, and emphasize the importance of considering potential effects (such as habituation) of human presence (or manipulations) in ecological studies, both in setting up experimental designs and reaching conclusions as to the questions initially addressed. Whereas habituation may be potentially beneficial to scientific research and tourist management, our study also raises the question of the potential influence of human activities on directional selection of specific phenotypes, and underlines the importance of physiological studies for appropriate conservation measures to be addressed [71].

## Authors’ contribution

VAV participated in study conception, analyzed the data and wrote the manuscript. ADS participated in data analyses and helped to draft the manuscript. BG performed the field work. RG conceived the study, participated in its design and coordination, participated in field work, and helped to draft the manuscript. All authors read and approved the final manuscript.
